# Early Autonomy May Contribute to an Increase in the General Surgical Workforce

**DOI:** 10.7759/cureus.7108

**Published:** 2020-02-26

**Authors:** Megan Quinn, Bracken Burns, Melissa Taylor

**Affiliations:** 1 Epidemiology and Public Health, College of Public Health, East Tennessee State University, Johnson City, USA; 2 Surgery, Quillen College of Medicine, East Tennessee State University, Johnson City, USA; 3 Surgery, The University of Texas Medical Branch, Galveston, USA

**Keywords:** physician shortages, general surgery, autonomy, resident education

## Abstract

Background: Nationally, 85% of general surgery graduates pursue fellowships reducing the incoming general surgical workforce with a predicted shortage of 41,000 general surgeons by 2025. In recent studies, the lack of confidence appears to be a major factor contributing to resident decision to pursue fellowship. The aim of the study was to determine if a hybrid academic/community program contributes to early autonomy and the decision to pursue fellowship in general surgery graduates. Methods: We evaluated the level of confidence, level of autonomy, and decision to pursue fellowship at a hybrid academic/community program that historically graduates 70% of their residents into general surgery practice through an anonymous survey. Participants responded using Likert scales along with simple polar questions. Results: Most current residents (90%) reported, upon graduation, that they feel very confident (45%) or fairly confident (45%) performing major cases independently. Most attendings (64%) reported that during their third year of residency, they began performing the majority (more than 75%) of their major cases as surgeon junior while current residents (55%) reported they were performing the majority as a second-year resident. Fifty-five percent of residents felt that confidence played a role in the decision to pursue fellowship. Thirty-three percent of our current chief residents and only 34% of the total general surgery residents plan to pursue fellowships.

Conclusions: Our study showed that our residents appear to have earlier levels of autonomy than that experienced by our practicing surgeons when they were residents. Confidence continues to play a role in the decision to pursue fellowship and overall our residents are confident in technical skills at graduation. Our unique program continues to graduate the majority of our surgical residents into successful general surgery practice.

## Introduction

The American surgical residency training model, established by Dr. William Halsted in the late nineteenth century, included graduated levels of responsibility. Recent changes in society have had effects on this system of training, hindering the progression toward autonomy in graduating surgical residents. A survey by Mattar et al. of fellowship program directors on the readiness of incoming fellows, stated that a strong foundation on graduation was needed to successfully perform complex cases independently by the completion of fellowship [1]. This paper concluded that 42% of fellowship directors felt that incoming fellows were unable to perform 30 minutes of a major procedure independently on entering fellowship and an astounding 56% were not proficient in laparoscopic suturing. Most directors commented that a lack of autonomy and independence during residency delayed progress in fellowship. In a 2015 study by Patel et al., program directors agreed that autonomy on surgical procedures was defined as completion of >75% of a case or the critical steps of a case and that most chief residents do not achieve complete autonomy [2]. These findings not only concern the progression during sub-specialization training programs but also on the readiness of graduates to pursue practice at the completion of residency. Current statistics show almost 85% of graduating general surgery residents proceed into fellowship resulting in only 200 graduates per year entering the general surgical workforce [3]. The Association of American Medical Colleges estimates a shortage of 41,000 general surgeons by 2025 [4]. One of the leading factors cited on resident decision to pursue fellowships is resident confidence [5]. East Tennessee State University inaugurated its general surgery residency program in 1978 and since then 72% of graduates practice broad-based general surgery [6]. While there have been variations between years, this number has remained consistent before and after the institution of duty hours. Created initially from legislation aimed at educating rural community providers, residents rotate at two level one private hospital, a 111 bed Veterans Affairs Medical Center, and a rural-based level two private medical center. Qualities regarding the hybrid academic/community program that contribute to resident decision to practice general surgery have yet to be identified. The objective of this study was to quantify levels of autonomy and confidence in surgical residents at our institution and attempt to describe factors that contribute to the decision to pursue general surgical practice.

## Materials and methods

An anonymous 38-question paper survey was distributed to current residents and attending surgeons at the four hospitals that comprise our training institution. Informed consent was obtained prior to voluntary participation. Attending surgeons were active practicing surgeons who provide direct resident education in their clinical practice. All resident surgeons were active in their clinical years. There were no research or off-cycle residents. The study received exemption from the East Tennessee State University Institutional Review Board (c0816.20e). The participants responded to simple polar, demographic, and five-point rating questions on a Likert scale. Survey responses were collected between September and October 2016. Descriptive statistics were calculated for quantitative responses.

## Results

There were 29/30 residents (97%) and 15/28 attending surgeons (54%) that responded with an overall response rate of 74%. Demographic data of the respondent attending surgeons is seen in Table 1. Of the respondent attending surgeons surveyed, 53% (8/15) graduated after duty hour institution. The majority of attending surgeons were university faculty (60%); 67% of responding surgeon faculty were fellowship trained. In contrast, 33% of our current chief residents and only 34% of the total general surgery residents at our program plan to pursue fellowships. 

**Table 1:** Respondent demographics

Table 1: Respondent Demographics	
	Respondent n (%)
Attending Surgeon	15 (34)
Years of Practice	
<5	3 (20)
5-10	5 (33)
11-20	2 (13)
>20	5 (33)
Type of Practice	
Hospital Employed	3 (20)
Private Practice	3 (20)
University Faculty	9 (60)
Fellowship Trained	
Yes	10 (67)
No	5 (33)
Resident Surgeon	29 (66)
Chief Resident	6 (21)
Plan for Fellowship	2 (33)
Plan for general surgery practice	4 (67)
All Residents	
Plan for Fellowship	10 (34)
Plan for general surgery practice	9 (31)
Undecided	10 (34)
*percentages derived from number of respondents for each question	

**Figure 1:**
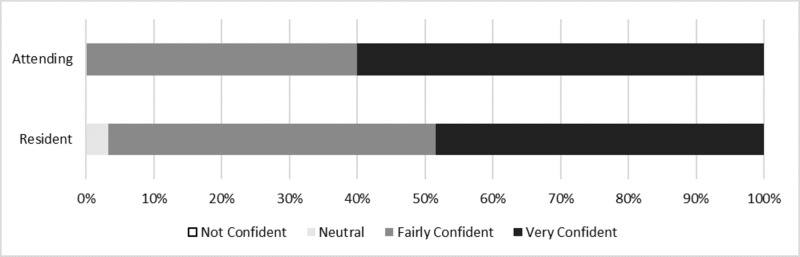
Confidence level in performing cases independently at the completion of a five-year general surgery residency

Residents and attending surgeons were questioned on their level of confidence in independently performing cases at graduation. Attendings reported that upon graduation from residency, they felt very or fairly comfortable performing cases independently (100%) and none reported feeling not confident. The majority of current residents (90%) reported upon graduation they will feel very confident (45%) or fairly confident (45%) performing major cases independently. The remaining 10% of residents were undecided or neutral. No residents reported they would not feel confident (0%) (Figure 1). Fifty-five percent of residents felt confidence played some role in the decision to pursue fellowship while only 41% felt student debt and potential income were a factor.

**Figure 2:**
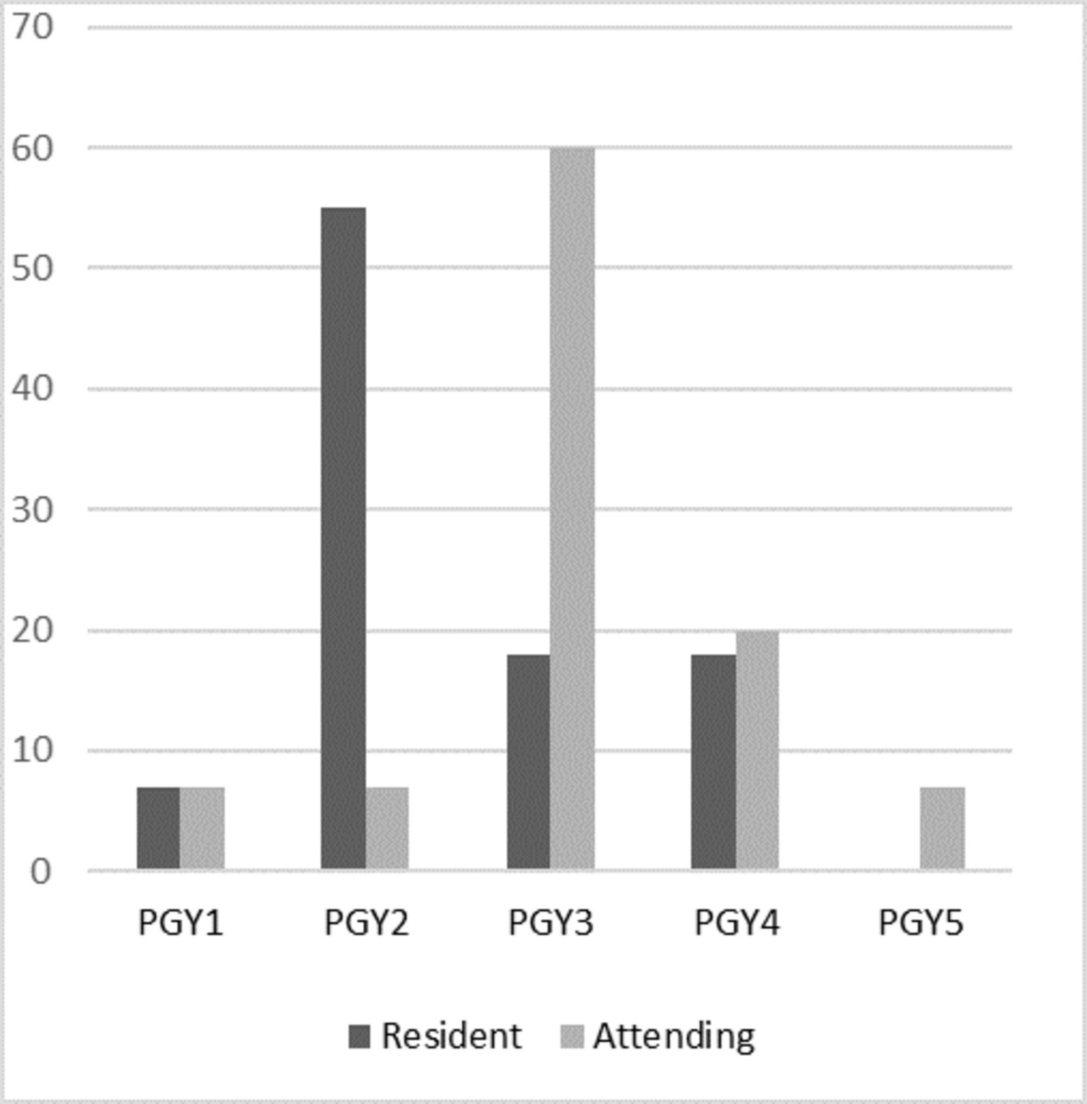
Level at which respondents felt they obtained surgeon junior* in greater than 75% of their major cases *Surgeon junior is completion of >50% of critical steps in the procedure.

**Figure 3:**
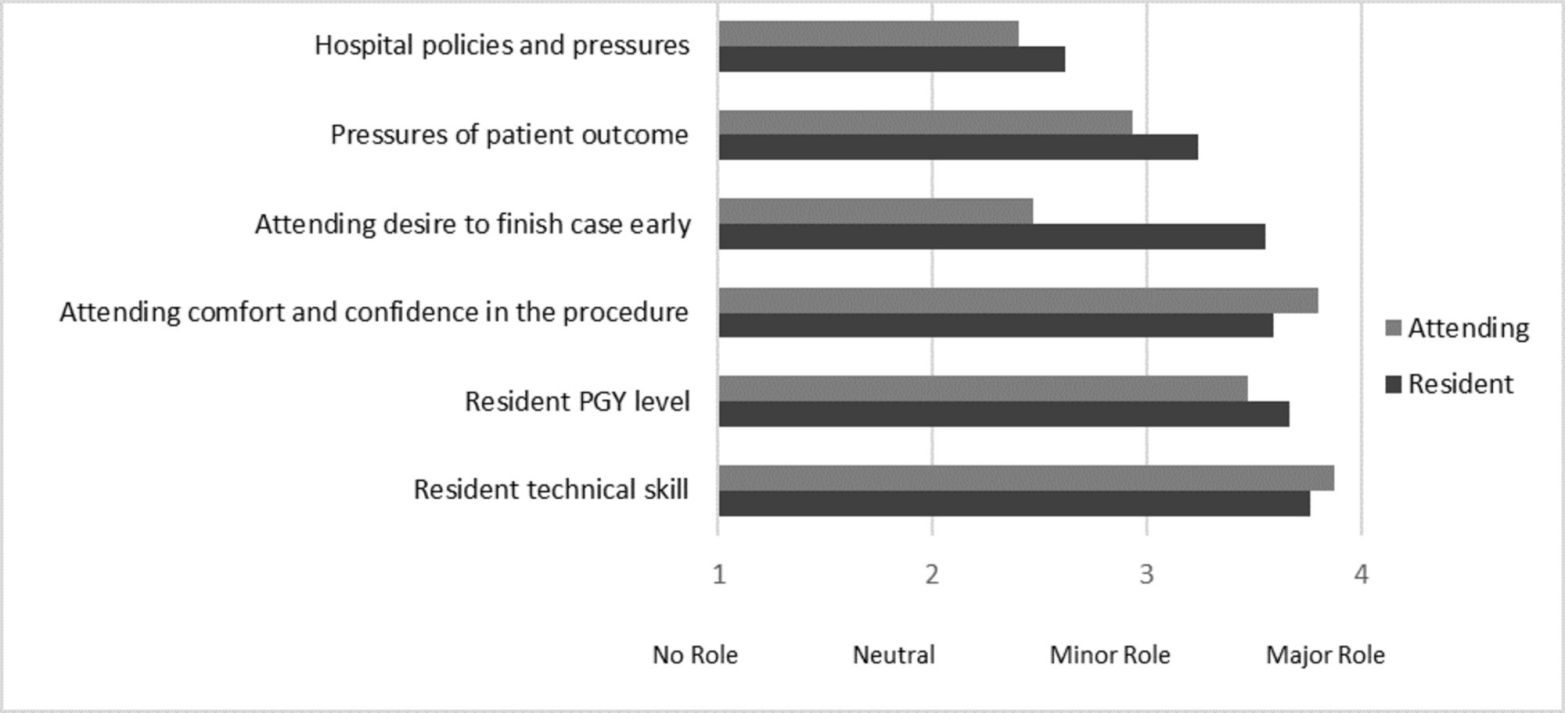
Mean responses regarding the role of listed factors in the level of autonomy given to a resident

Most attendings (64%) began performing more than 75% of their major cases as surgeon junior (>50% of the case performed independently) as a third-year surgical resident, while current residents (55%) reported they were performing >75% of their major cases as surgeon junior as a second-year resident (Figure 2). Resident and attending surgeons were inquired regarding factors that contribute to the amount of autonomy a resident is given during a procedure (Figure 3). Resident technical skill overall was the most deciding factor (82% of all respondents felt it was a major factor) followed by attending comfort and confidence in the procedure (73%) and resident postgraduate level (70%). Residents appeared to feel attending desire to finish quickly (62% felt it was a major factor) and pressures of patient outcome (59%) were more of a factor than attending surgeons (7% and 20% respectively). Only 18% of all respondents felt hospital policies and pressures played a major role.

## Discussion

While many of our residents continue to pursue general surgery practice after graduation, confidence appears to play a major role in the decision to pursue fellowship. This suggests that our residents are overall confident in their ability to practice broad based general surgery independently at graduation. We looked at factors that may contribute to this level of confidence through an anonymous survey of active attending surgeons and current residents. 

Fitts and Posner’s Three Stages of Learning is an established model of acquiring skills during surgical training of residents described in the literature [7]. It divides the learning of a skill into cognitive, integrative and autonomous stages. A skill first must be understood, in the learning stage, by breaking it down into the fundamental steps. By the integrative stage, the learner is able to complete a skill but still requires active alertness during each step of the task. As the learner reaches the autonomous stage, the skill becomes second nature, a task that does not require active thinking to complete and allows for precision and adaptability. A 2014 survey of attending surgeons found that most felt current residents have less operative autonomy, less exposure to the operating room, graduate with less cases, are less ready to operate independently, and have less broad based general surgery knowledge [8]. Many curriculum models developed in the last few years aim at assisting in the progressive development of autonomy including the Accreditation Council for Graduate Medical Education (ACGME) milestones, entrustable professional activities (EPAs) and the Zwisch model. Resident respondents of our survey were completing >75% of their cases as surgeon junior at an earlier training level than that experienced by attending surgeons as residents. Respondent residents and attending surgeons both concluded that the level of autonomy is most often dictated by the resident technical skill, post graduate level, and attending confidence in the procedure as previously seen in literature [8]. The current curriculum at our institution is guided by the ACGME milestones and designed with an emphasis on broad-based experiences including vascular surgery, gastrointestinal/oncologic surgery, and advanced laparoscopic surgery [6]. 

A 2017 study by George et al. of 14 general surgery residency programs found that for core procedures performed in the last six months of surgical residency, a Zwisch level of near independence (no help, supervision only), only 33.3% was attained [9]. Many will argue that this is insufficient for the transition to general surgery practice. Our study showed that current residents at our program were overall confident of their ability to practice independently at the completion of residency. Residents rotate on services between four different hospitals where there are no sub-specialty surgical training fellows. Additionally, the program is one in only 10% of all surgical training sites that have ACGME accredited international surgical experience. International rotations benefit residents by learning resource utilization, cultural competency, and exposure to advanced pathology [10]. These opportunities in training for our residents expose them to not only more broad exposure but additionally more complex cases and clinical decision making which would otherwise be distributed to specialty fellows. With a stable 1000 surgeon graduates per year and 85% of them specializing, the incoming general surgeon workforce is reduced to approximately 200 per year [3]. Hospitals need general surgeons to respond to surgical and traumatic emergencies. Inadequate on-call surgeon coverage is reported in 75% of hospitals [4]. A smaller general surgeon pool endangers the rural and suburban hospitals as without general surgeons, they may be forced to close down. Our hybrid academic/community general surgery training program is unique in contributing 70% of our graduates into the general surgical workforce. Our residents agree with national studies that confidence is a factor in the decision to pursue fellowship however the trend towards chief level is a decreasing contribution to the decision. Student debt and income potential were less of a factor. This suggests other factors such as lifestyle and mentorship as potential contributors. Access of rural areas in the United States to a general surgeon is in crisis. Congress introduced a bill in 2017 “Ensuring access to General Surgery” to investigate these gaps [11]. There has only been a modest (7.5%) increase in categorical surgical positions since 1990 despite population growth. A 2003 study estimated there would be a 31% increase in surgical services between 2001 and 2020 secondary to an expanding and aging population resulting in a 9% shortage in the surgical workforce [12]. Etzioni et al. also estimated that if an immediate 15% increase in residency training positions occurred, after 20 years only an increase of 7.5% of trained general surgeons would be seen [13]. Without an immediate effective change in general surgical residency training models to provide confident and competent young practicing general surgeons, incentives to practice rural general surgery, and movement to make the practice attractive to graduating medical students, the supply of general surgeons in America is going to rapidly decline in relation to the growing population. Our study showed a consistent 67% of residents at our institution pursuing general surgery practice after graduation and overall our residents felt confident regarding the ability to operate independently at the completion of residency. Limitations of the study include single-institution analysis with a small sample size limiting the statistical evaluation. Analysis of the complexity of the procedures at which residents felt they achieved as surgeon junior would be interesting to compare with autonomy levels measured in newer quantifiable training evaluation methods. We would also like to see levels of confidence as each class graduates in the level of training and re-evaluate graduating chiefs after they have started their practice. Despite these limitations, an earlier level of autonomy seen in our residents likely plays a crucial role in overall confidence, attracting residents to pursue general surgical practice and incentive for applicants to consider our program. Early autonomy should be expressed as a critical component in resident education and success. National measures to help support the role of autonomy in resident education while not compromising patient safety is a topic of needed research.

## Conclusions

Our study showed that our residents appear to have earlier levels of autonomy suggesting a role in greater confidence of residents on completion of general surgery training. Our program continues to graduate the majority of our surgical residents straight into practice, contributing to the general surgical workforce.
